# Multi-trait phenotypic modeling through factor analysis and bayesian network learning to develop latent reproductive, body conformational, and carcass-associated traits in admixed beef heifers 

**DOI:** 10.3389/fgene.2025.1551967

**Published:** 2025-03-24

**Authors:** Muhammad Anas, Bin Zhao, Haipeng Yu, Carl R. Dahlen, Kendall C. Swanson, Kris A. Ringwall, Lauren L. Hulsman Hanna

**Affiliations:** ^1^ Department of Animal Sciences, North Dakota State University, Fargo, ND, United States; ^2^ Department of Statistics, North Dakota State University, Fargo, ND, United States; ^3^ Department of Animal Sciences, University of Florida, Gainesville, FL, United States; ^4^ Dickinson Research Extension Center, North Dakota State University, Dickinson, ND, United States

**Keywords:** Bayesian network, latent phenotypes, multi-trait modeling, factor analytic models, heifer development, phenomics

## Abstract

Despite high-throughput and large-scale phenotyping becoming easier, interpretation of such data in cattle production remains challenging due to the complex and highly correlated nature of many traits. Underlying biological traits (UBT) of economic importance are defined by a subset of easy-to-measure traits, leading to challenges in making appropriate selection decisions on them. Research on UBT in beef cattle is limited. In this study, the phenotypic data of admixed beef heifers (n = 336) for reproductive, body conformation, and carcass-related traits (traits, t = 35) were used to identify latent variables from factor analysis (FA) that can be characterized as UBT. Given sample size constraints for carcass (n = 161) and other body size-related traits (n = 336), two models were explored. In Model 1, all individual traits were considered (n = 161), while in Model 2, the dataset was split into body size (n = 336) and carcass (n = 161) traits to maximize available heifers per dataset. A combination of FA and Bayesian network (BN) learning was adopted to develop UBT and infer BN structure for subsequent analyses. All heifers (n = 336) were genotyped using GeneSeek Genomic Profiler 150K for Beef Cattle. Following quality checks, 117,373 autosomal SNP markers were retained and used for genomic estimated breeding values (gEBV) in BN learning steps. Using exploratory and confirmatory FA, Body Size (BS) and Body Composition (BC) were identified as UBT for Model 1, explaining 14 phenotypic traits (t = 14). For Model 2, BS, Ovary Size, and Yield Grade (YG) were identified as UBT, explaining 12 phenotypic traits (t = 12). When using gEBV, the causal network structure inferred showed BS contributed to BC in Model 1 and to Ovary Size in Model 2. Therefore, a structure equation-based approach should be used in subsequent modeling for these traits. From Model 2, YG should be modeled univariately. This study is the first to identify UBT in growing admixed heifers using body size, conformation, and carcass traits. We also identified that BC and YG did not explain intra-muscular fat and body density, indicating these two traits should also be modeled univariately.

## 1 Introduction

Development of high throughput genotyping technologies paved the path for phenome to genome translation, but challenges in obtaining economically important phenotypes are still present (Yu et al., 2020b). Advanced phenotyping technologies present opportunities to obtain these phenotypes, but they also introduce challenges in animal selection given the amount of data collected and interpreted in terms of related phenotypes (Silva et al., 2021). Single trait-based estimation of breeding values (EBV) either through pedigree records or estimation of genomic EBV (gEBV) through genome-wide studies are well-established (Guo et al., 2014). However, due to increased phenotypic data complexity, multi-trait genome-wide modeling using gEBV may be necessary to handle associations considering interrelated traits (Henderson and Quaas, 1976; Guo et al., 2014). The multi-trait EBV approach, as explained by Henderson and Quaas (1976), treats each trait as a separate measure in the selection model. This can be problematic when variables explained by the same underlying phenotypic trait are included, potentially causing the model to overfit. Due to this, not all variables can be included as a separate measure in the model, thereby demanding a different strategy to handle their correlated nature.

The concept of an underlying biological trait (UBT), a hidden trait representing a set of interrelated traits, is becoming more frequent due to the development of robust interrelated phenotypic measures captured through technological advancement (Mccormick et al., 2016). The UBT are termed as a combined influence of observed phenotypic traits but there is no real structure to find out the contributing phenotypic traits making these traits biologically meaningful (Olasege et al., 2019). For example, a recent approach to handling interrelated linear traits in Holstein cattle has used 9-point scales for traits such as body conformation, foot and leg conformation, udder conformation, dairy capacity, and total score (Čítek et al., 2022) in multi-trait modeling of these composite traits (Lu et al., 2018). Even so, these are subjective scales captured holistically rather than by individual trait attributes, therefore the understanding of their biological correlation can be influenced strongly by the evaluator. Therefore, this approach is prone to error and likely inconsistent in application across populations based on evaluator experience. To minimize subjectivity and adopt a better data-driven approach to explain the structure of interrelated underlying phenotypic traits, factor analysis can be a way forward (Campos et al., 2012; Olasege et al., 2019). Factor analysis is a computationally efficient data-driven approach to investigate possible latent variables, leading to the development of UBT when individual and correlated traits are available (Olasege et al., 2019), but its application in livestock, especially cattle, remains limited (Yu et al., 2020a; Pegolo et al., 2021).

Factor analysis initially was developed by psychologists to identify interrelated measurements (Vincent, 1953) and later for data reduction (Tavakol and Wetzel, 2020). This approach has also been adopted in other fields such as plant sciences for improved selection decisions. Factor analysis efficiently explains correlations among interrelated traits, leading to biologically and economically relevant UBT (Toker and Ilhan Cagirgan, 2004). Factor analysis was also adopted for improved genome-wide association of complex traits in dairy (Macciotta et al., 2012; Lurdes Kern et al., 2014) and dual-purpose cattle breeds in China (Xu et al., 2022). Exploratory factor analysis (EFA), as introduced by Hartley (1954), identifies a method to learn about the pattern present in interrelated traits, whereas confirmatory factor analysis (CFA) assumes there is previous knowledge of possible UBT. It is rare to start directly with CFA in most cases, therefore EFA is used initially.

To ensure the appropriate application of UBT in genome-wide analysis or genetic evaluation models, a joint application of factor analysis and Bayesian network learning (BNL) approaches was recently demonstrated in wheat yield and pathophysiology data (Momen et al., 2021), and agronomic traits data in rice (Yu et al., 2019). Exploratory factor analysis, previously known as inferential factor analysis, is used to infer underlying hidden latent variables and demands no previous knowledge of variable structure (Haig, 2018). The EFA is then followed by CFA to refine the variable structure provided by EFA and to develop values of the UBT for subsequent analyses. The CFA output is followed by BNL to infer the potential causal network structure(s) among latent traits (Momen et al., 2019; Yu et al., 2020a). This approach has been adopted in plant models such as rice (Yu et al., 2019) and wheat (Momen et al., 2021), in dairy cattle for genome-wide association of milk protein fraction (Pegolo et al., 2021), and in beef cattle to compare temperament scoring methods (Yu et al., 2020a). Even so, further exploration of this approach is still warranted, especially by adjusting it with genomic data in cattle. The directed trait network developed through BNL is adjusted based on genomic breeding values that are transformed to be uncorrelated to avoid false network structure, the primary assumption of a Bayesian network (Töpner et al., 2017; Yu et al., 2019).

A unique research population of admixed beef cattle available through the North Dakota State University (NDSU) Dickinson Research Extension Center (DREC) provides an opportunity to explore the use of EFA and BNL on a combined dataset of reproductive, body conformation, and carcass composition measures collected at yearling age. The interrelated nature of these measures is unclear and not reported previously; therefore, this dataset provides an opportunity to explore the latent structures and networks present in growing admixed beef heifers to better design genome-wide modelling. Objectives of this study are 1) to identify possible latent traits underlying reproductive, body conformation, and carcass composition measures collected from admixed beef heifers using exploratory and confirmatory factor analysis and 2) to identify interrelations among these latent traits by including the genomic breeding values in the BNL structure. The latent traits identified are expected to explain most of the variation of the associated traits, developed UBT, and identify their interrelationship for subsequent genome-wide studies modelling.

## 2 Methods

All procedures involving data collection were approved by the NDSU Institutional Animal Care and Use Committee (IACUC reference No. A15062 and A18065). Data used in this study were sourced from yearling admixed beef heifers (n = 336, average age of 12.71 ± 0.51 months) produced at NDSU DREC and who completed a feed trial at the NDSU Beef Cattle Research Complex (BCRC). This population was previously described by Bhowmik et al. (2023), where heifers considered for this study are daughters of the admixed base herd born from 2014 to 2017 (n = 254) along with their subsequent daughters born in 2016 and 2017 (n = 81). Based on pedigree and following Bhowmik et al. (2023), primary breed types of heifers considered in this study included influenced (I, ≥50%) of American Aberdeen (ADI; n = 59), Angus (ANI, black Angus; n = 42), Red Angus (ARI; n = 136), Gelbvieh (GVI; n = 15), Limousin (LMI; n = 4), Shorthorn (SHI; n = 10), and Simmental (SMI; n = 35), as well as true first crosses of available purebreds (F_1_; n = 33). Final sample sizes overall and by primary breed type depended on traits considered and available records.

### 2.1 Phenotypic data pre-processing

**FIGURE 1:**
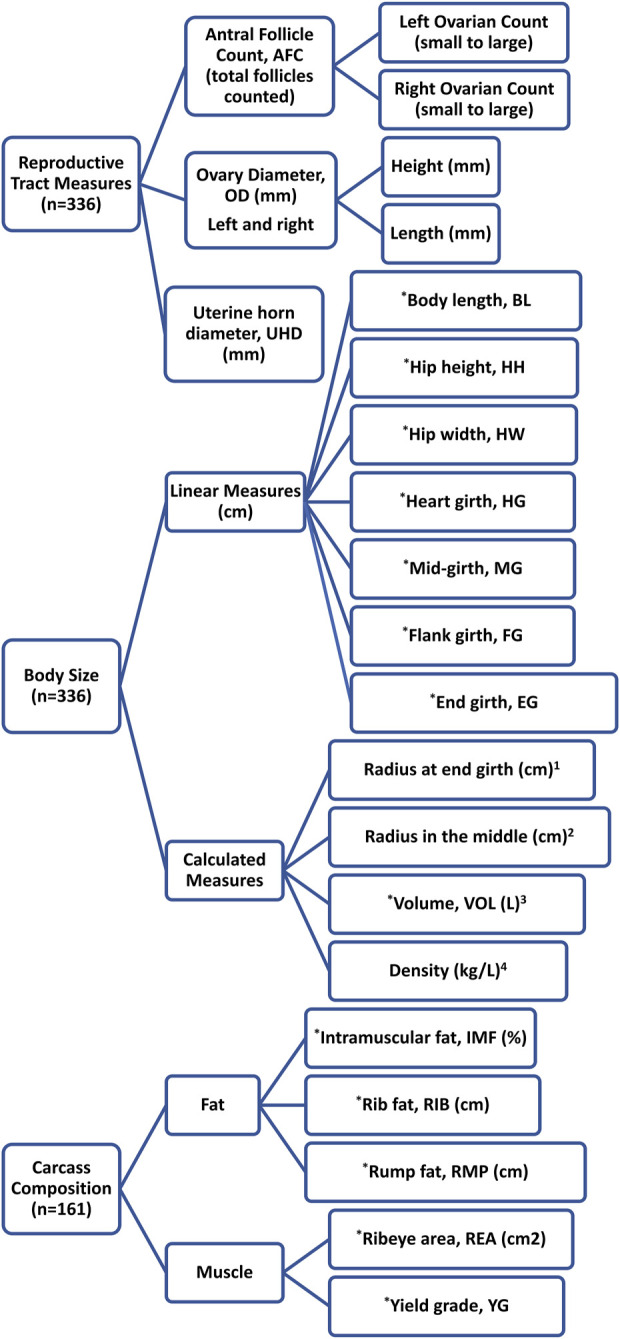
Phenotypic measurements (direct and calculated) of yearling beef heifers available for use in exploratory factor analysis. ^*^Initial, final, average, and daily gain measurements were recorded for these parameters but only initial measurements were used for the rest of the analyses; ^1^Radius at end girth = end girth/2π; ^2^Radius in the middle = mid-girth/2π; ^3^Volume (L) = ((π × body length × (radius at end girth ^∧^2 + (2 × radius in the middle ^∧^2)))/3)/1000; ^4^Density (kg/L) = body weight/volume (L).

Phenotypic measurements were recorded to explore UBT for reproductive performance, body conformation, and carcass composition. The heifer calves included in the data collection were born and raised at the NDSU DREC ranch near Manning, ND. The animals underwent a 105-day feeding period at the NDSU BCRC in Fargo, ND before and during their first breeding season. The data available for heifers were collected year-wise as 89, 73, 100, and 72 heifers from 2015 to 2018, respectively. All reproductive tract measurements (see Figure 1) were collected from all heifers at the start of the feeding period (n = 336 heifers) according to the protocol described by Cushman et al. (2009), while body conformation traits characterizing body size were measured at the start and end of the feeding period using a measuring tape or ultrasound equipment. The data associated with an average daily gain were calculated by taking differences in initial and final weight divided by the number of days in the trial (Bhowmik et al., 2023). A total of 161 heifers had ultrasound carcass measurements (see Figure 1) specifically from 2014 to 2015 born heifers. The ultrasound measurements were recorded following Wall et al. (2004) using an Aloka500V equipped with a 3.5-MHz linear array transducer (Corometrics Medical Systems Inc., Wallingford, CT). In addition to multiple phenotypic traits per heifer, most traits also had repeated measurements and averages of repeated measures available (see Figure 1). Considerations were placed with how repeated measures were used so that each timeframe across traits were aligned appropriately and modeled separately of other timeframes to avoid confounding data series. Due to this, it was found that body size measures in 2014-born heifers had errors associated with final feeding period measures that could not be resolved, therefore only initial measures were used for further analyses for these heifers relative to body size and carcass traits as well.

The phenotypic data recorded were then filtered for missing values and outliers, where data points were considered outliers based on limits described by Tukey’s outlier rule (Iglewicz, 2011). After filtering for outliers and missing values, traits with correlation (Pearson correlation, *r* ^2^ ≥ 0.85) and sample adequacy (measure of sample adequacy, MSA <0.5) were removed to ensure variables used in EFA would provide the best outcome. Filtering traits prioritized measures captured on the animal over calculated measures given the applicability and feasibility of producer implementation while also following recommendations by Akoglu (2018).

### 2.2 Exploratory factor analysis (EFA)

Following approaches established by Momen et al. (2021) for EFA, the R v4.1.3 package *psych* v2.3.3 (Revelle, 2017) was used to identify the hidden latent factors as complex underlying biological phenotypes, accommodating the effect of multiple traits (see Figure 1). Sample adequacy is assessed by the Kaiser-Meyer-Olkin criterion, which ranges from 0 to 1. Adequate datasets should fall between 0.5 and 1 to ensure factor analysis can proceed (Kaiser, 1958), therefore a threshold of 0.5 was used to measure sample adequacy (MSA) in this study. The factor retention decision for EFA procedures was made based on parallel analysis using 1,000 simulations per group of traits analyzed (Horn, 1965; Hayton et al., 2004).

### 2.3 Confirmatory factor analysis (CFA) and bayesian network learning (BNL)

The downstream confirmatory factor analysis (CFA) was performed based on the EFA latent structure for each latent trait using *blavaan* v0.4-7 (Merkle and Rosseel, 2015) package in R with priors set to default values. The CFA approach was adopted to refine the variable structure and to further remove the cross-loading of latent factors provided by EFA. The Markov chain Monte Carlo (MCMC) sampling method employed in *blavaan* was set to 10,000 iterations, where 5,000 samples were retained in all given analyses after discarding the first 5,000 as burn-in samples to meet the convergence criteria. The convergence criteria of the model were diagnosed by calculating posterior standard deviations of the model, where a value at or close to 1 indicated convergence was achieved (Gelman and Rubin, 1992). The coefficient of determination (*R* ^2^) for each trait in the model was also estimated to see how much these values align with factor loading criteria, defining the latent variable structure for underlying biological phenotypes. The posterior mean values of the model were then assigned to UBT as a new phenotype score for BNL.

The Bayesian network is a directed graphical representation of conditional independence of random variables (Heckerman et al., 1995). In this study, we applied a score-based algorithm, Tabu, and a hybrid algorithm, Max-Min Hill Climbing, to identify underlying latent traits’ network at the genetic level using the *bnlearn* v4.8.1 R package (Scutari and Denis, 2021). The latent variables produced from CFA in both models were then processed in Bayesian network learning algorithms to identify latent variable interrelationships per model. The network directional uncertainty and strength were measured through bootstrapping (Scutari and Denis, 2021).

### 2.4 Genomic data processing to adjust BNL structure

The BNL structure inferred based on estimated latent traits may not be accurate and requires adjustments for the gEBV to remove the confounding effects within traits and to model them either through univariate, multivariate, or structure equation-based genome-wide association analysis (Töpner et al., 2017; Yu et al., 2019). To get the genotypic relationship matrix, the initial population of heifers (n = 336) was genotyped using the GeneSeek Genomic Profiler 150K for Beef Cattle (Neogen GeneSeek, Inc., Lincoln, NE) and quality checked (QC) as described by Bhowmik et al. (2023). Briefly, genotyping resulted in 138,893 SNP markers, but only 132,368 autosomal SNP markers were further analyzed for this study. The QC criteria of minor allele frequency (MAF) threshold of 5%, call rate of markers more than 95%, and an exact Hardy-Weinberg equilibrium (*P* < 0.0001) (Wigginton et al., 2005) resulted in 117,373 SNP markers. All these QC analyses were conducted through R v4.1.3 and markers were retained for further analysis. The relationship matrix generated using *AGHmatrix* v2.1.4 (Amadeu et al., 2023) package of R was then used in multi-trait mixed model as random effect along with fixed effects of data collection year, dam-age, breed-influenced group, and generation of heifers to estimate gEBV. The generated gEBV were further adjusted to remove the sample dependencies and then used as adjusted input for the BNL network structure (Töpner et al., 2017; Momen et al., 2021).

## 3 Results

### 3.1 Data pre-processing

The phenotypic data in this study were processed into two models given unsupervised (Model 1) and semi-supervised (Model 2) data-driven approaches. For Model 1, heifer data (n = 336, t = 35) were pre-processed for missing data and outliers given Tukey’s formula (Iglewicz, 2011), leaving us with 159 heifers (t = 35). After filtering for outliers and missing values, the highly correlated traits (Pearson correlation, *r* ^2^ ≥ 0.85; see Supplementary Tables S1–S3) were removed. Additionally, traits whose effects were explained in calculated measures like AFC (sum of all small, medium, large, and extra-large follicular count), LOD (average of left ovarian length and height), and ROD (average of right ovarian length and height), density (covers VOL effect) were also excluded to have enough sample adequacy as explained later in exploratory factor analyses. For Model 2, after filtering, the reproductive and body size datasets were left with a sample size of 298 heifers, while for carcass composition there were 161 heifers remaining. Considering this, two models were run, Model 1 with reproductive, size, and carcass data (n = 159, t = 16, overall MSA = 0.63) and Model 2 with the split dataset (i.e., reproductive and body conformation-only data, n = 298, t = 11, overall MSA = 0.57; and carcass composition data, n = 161, t = 5, overall MSA = 0.59).

### 3.2 Exploratory factor analysis (EFA)

**FIGURE 2:**
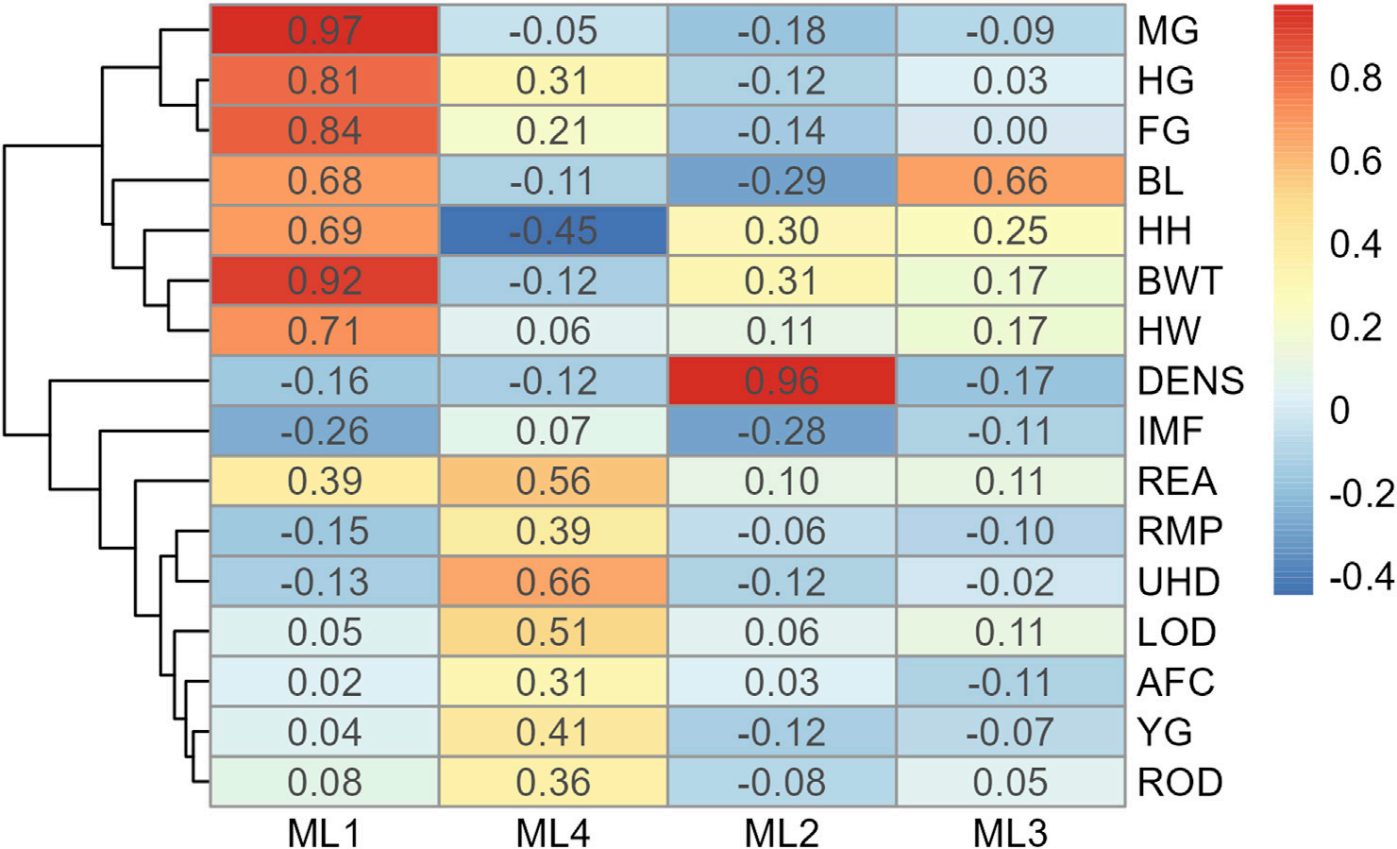
Heatmap of factor loading scores for Model 1 (all adequate variables) identifying latent variables (ML 1–4) from exploratory factor analysis. Abbreviations: MG, mid girth; HG, heart girth; FG, flank girth; BL, body length; HH, hip height, BWT, body weight; HW, hip width; DENS, body density; MF, intramuscular fat; REA, rib eye area; RMP, rump fat; UHD, uterine horn diameter; LOD, left ovary diameter; AFC, antral follicular count; YG, yield grade; and ROD, right ovary diameter.

**FIGURE 3:**
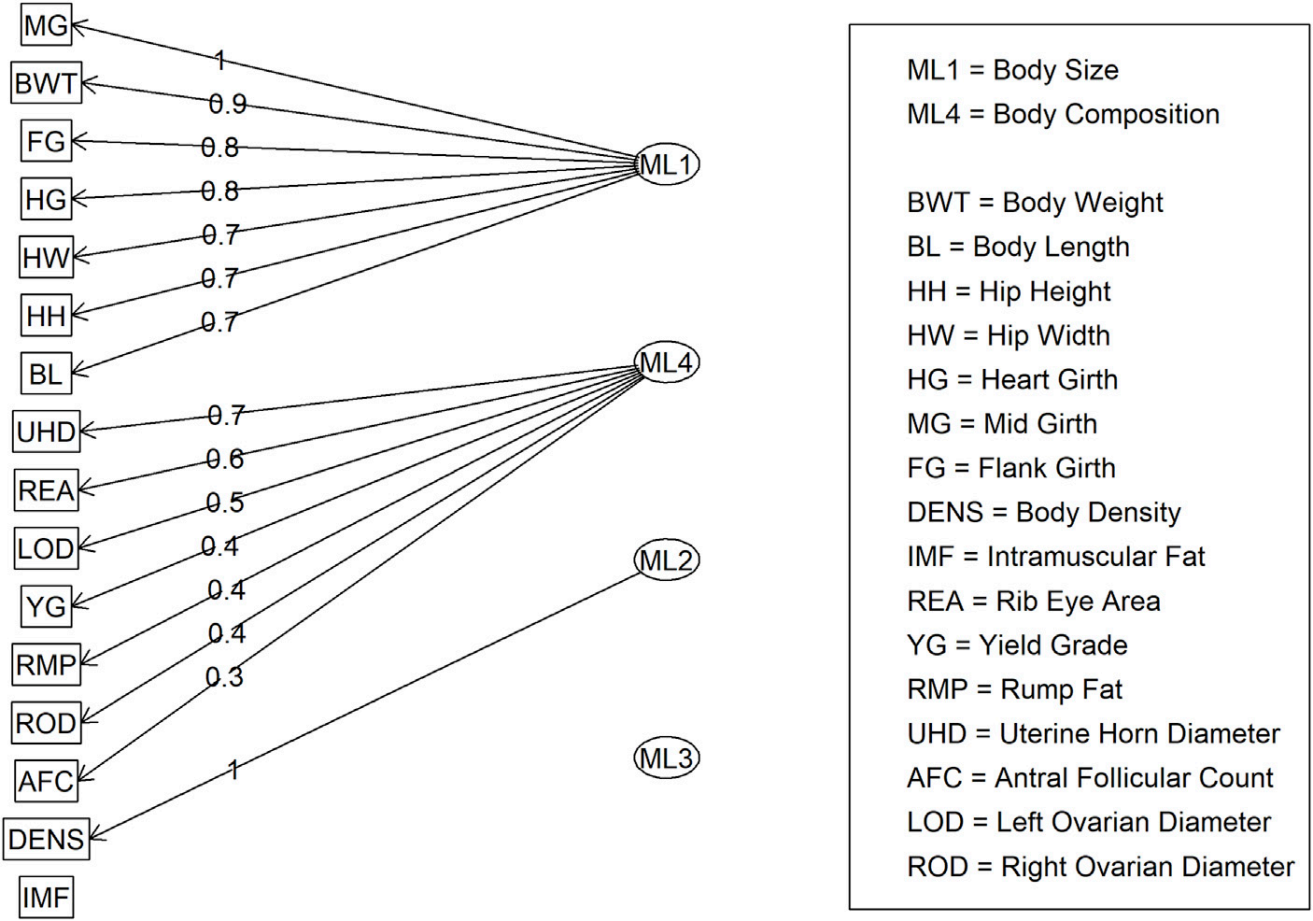
Factor diagram for Model 1 (all adequate variables) when assigning traits to their highest loading latent variable (ML).

For Model 1, factor loading values are shown in Figure 2. Some variables, such as ribeye area and body weight, contributed to multiple factors: ribeye area to factors 1 and 4, and body weight to factors 1 and 2. The latent variable structure was refined by a factor diagram, restricting the assignment of variables to the factor with the highest loading values when present in multiple latent variables (Figure 3), efficiently handling cross-loading. The EFA approach for Model 1 identified two latent variables of interest, which included body size (ML1 as BS; Figure 3) and body composition (ML4 as BC; Figure 3). These two latent variables were used in subsequent steps. The intramuscular fat (IMF) and body density (DENS) variables were not associated with either of these two latent variables when the EFA network was refined through CFA.

**FIGURE 4:**
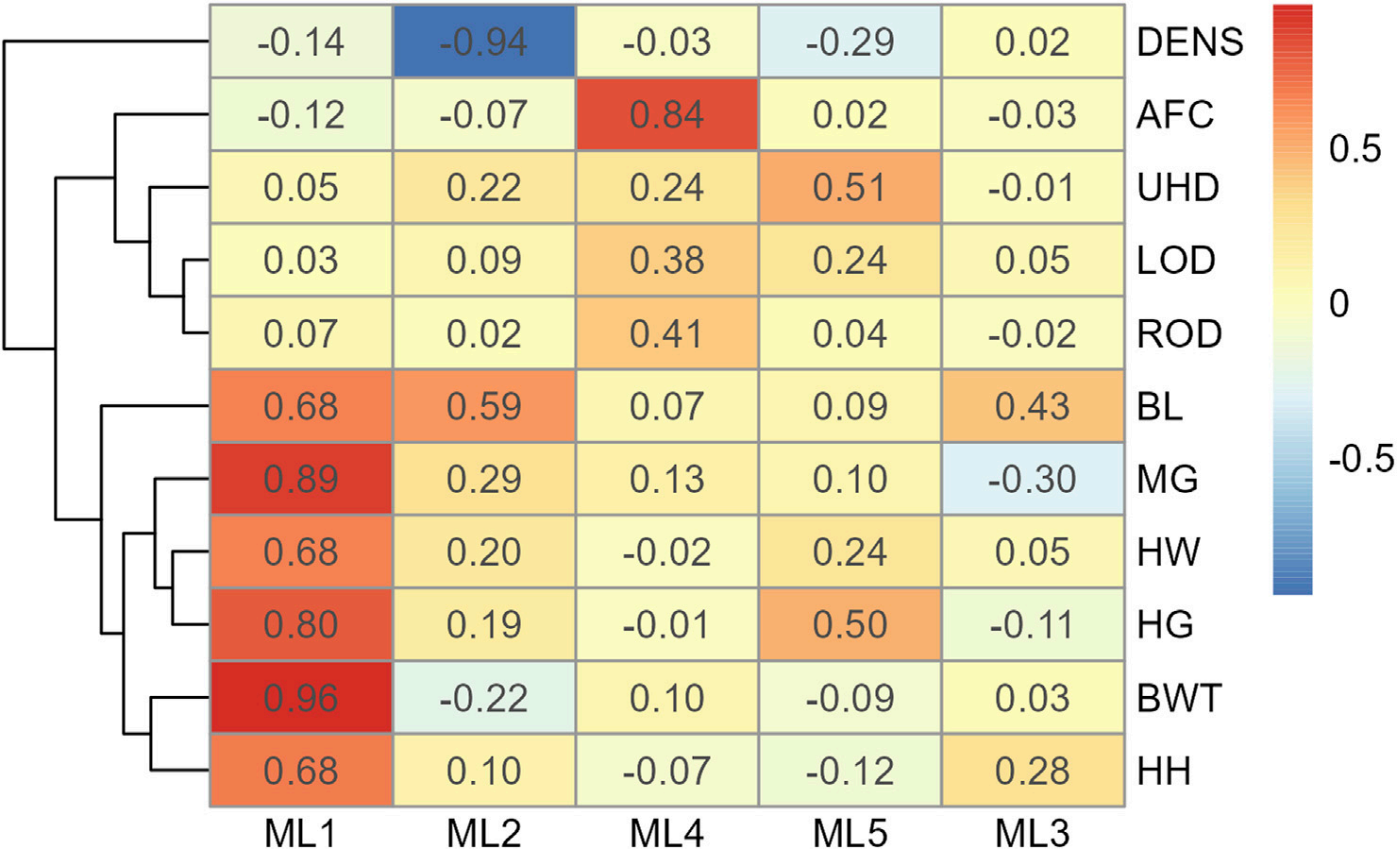
Heatmap of factor loading scores for Model 2 combine data for body conformation and ovarian traits identifying latent variables (ML 1–5) from exploratory factor analysis. Abbreviations: DENS, body density; AFC, antral follicular count; UHD, uterine horn diameter; LOD, left ovary diameter; ROD, right ovary diameter; BL, body length; MG, mid girth; HW, hip width; HG, heart girth; BWT, body weight; and HH, hip height.

**FIGURE 5:**
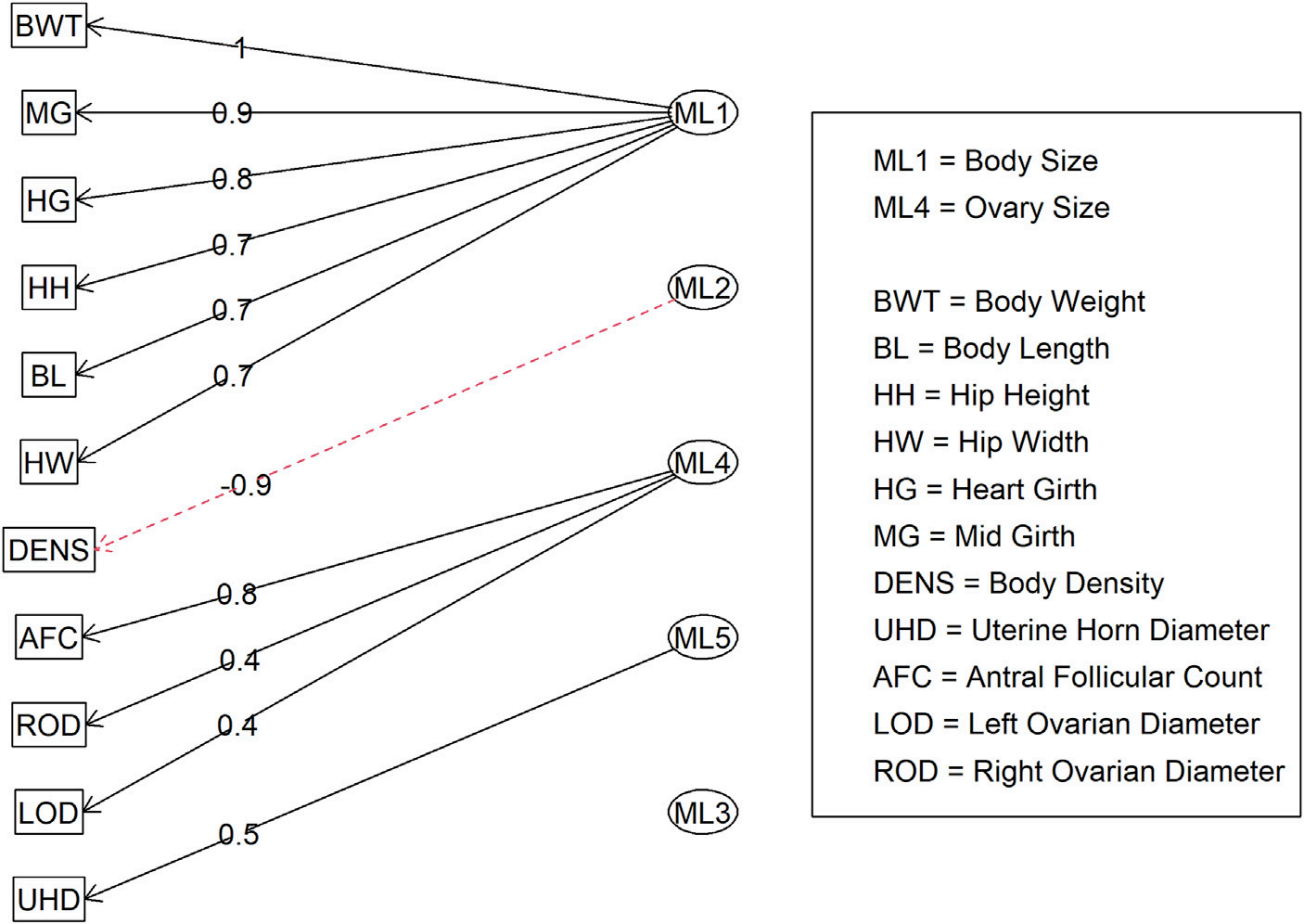
Factor diagram for Model 2 (combined body conformation and ovary traits dataset) when assigning traits to their highest loading latent variable (ML). Abbreviations: BWT, body weight; MG, mid girth; HG, heart girth; HH, hip height; BL, body length; HW, hip width; DENS, body density; AFC, antral follicular count; ROD, right ovary diameter; LOD, left ovary diameter; and UHD, uterine horn diameter.

**FIGURE 6:**
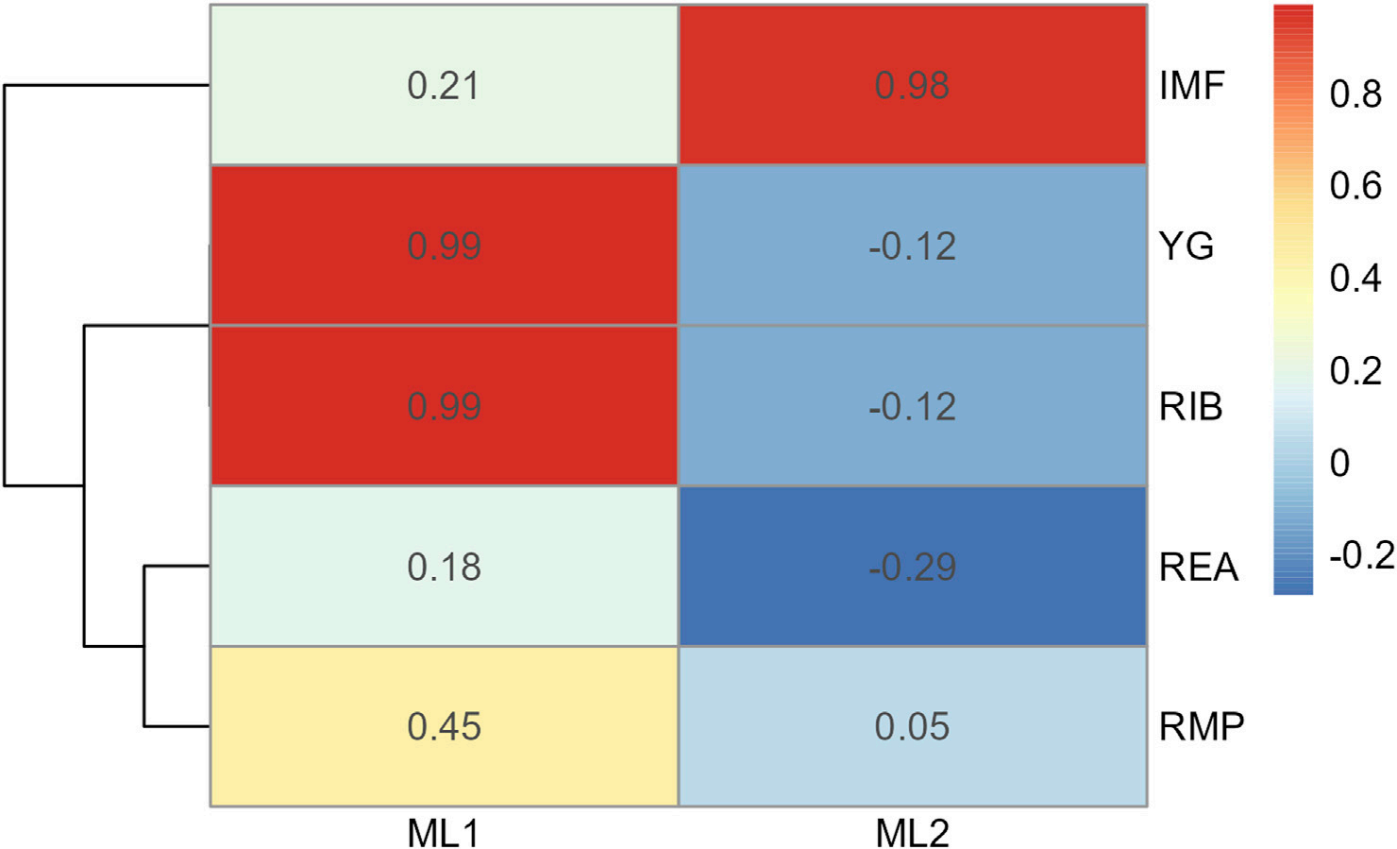
Heatmap of factor loading scores for Model 2 carcass only dataset identifying latent variables (ML 1–2) from exploratory factor analysis. Abbreviations: IMF, intramuscular fat; YG, yield grade; RIB, rib fat; REA, ribeye area; and RMP, rump fat.

**FIGURE 7:**
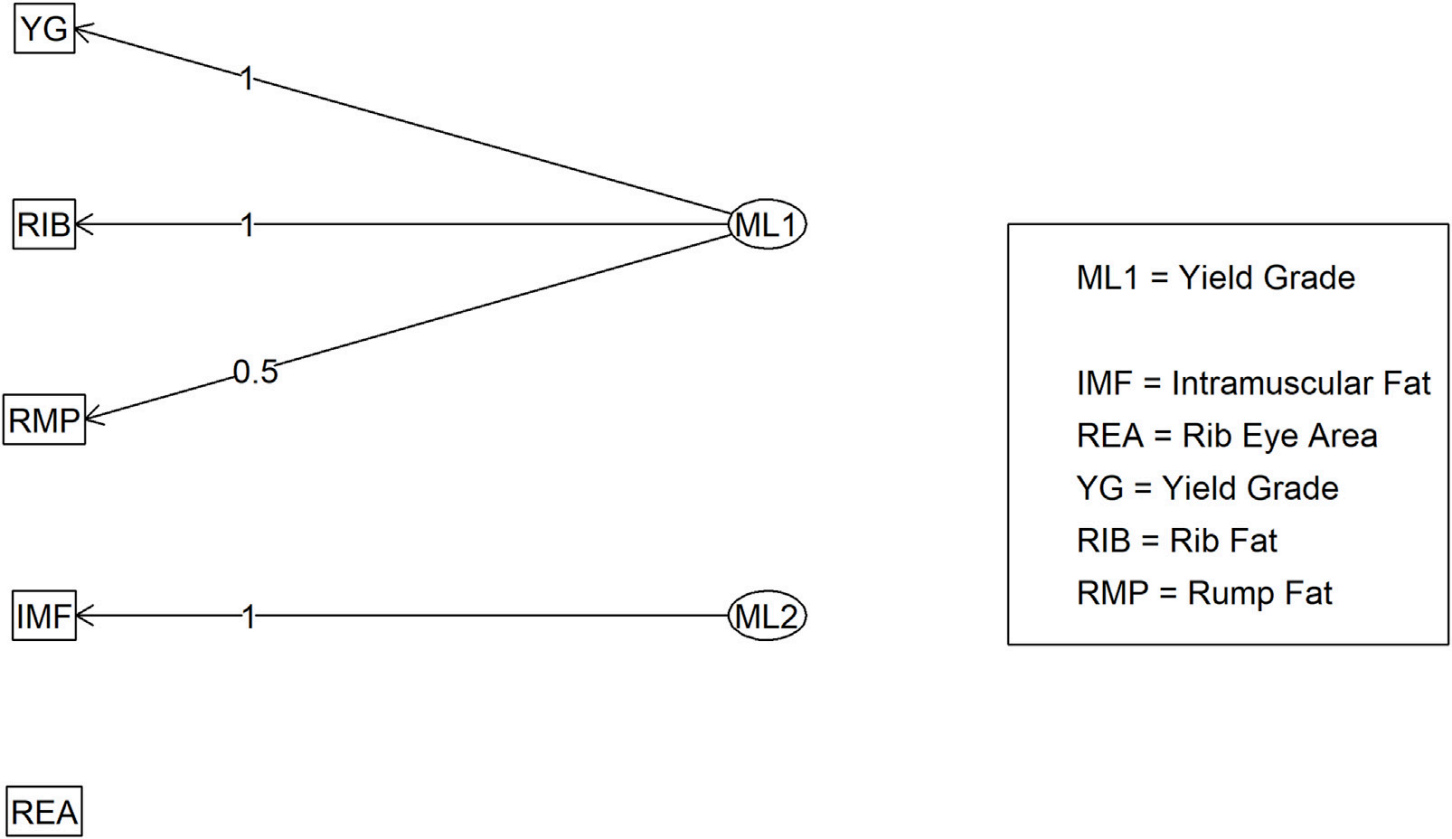
Factor diagram for Model 2 (carcass only dataset) when assigning traits to their highest loading latent variable (ML). Abbreviations: IMF, intramuscular fat; YG, yield grade; RIB, rib fat; REA, ribeye area; and RMP, rump fat.

In Model 2, the first EFA was performed for a combine dataset of body conformation and ovarian traits. The factor loading values for the first EFA of Model 2 are shown in Figure 4. As with Model 1, some variables had sufficient loading scores for more than one variable (i.e., body length was common to latent factors 1, 2, and 3, and heart girth was common to factors 1 and 5). The factor diagram shown in Figure 5, displays the latent variable with each variable’s highest loading score. Proceeding with the carcass-only dataset of Model 2, Figure 6 shows the factor loading scores from the second EFA, and the factor diagram of latent variables with each variable’s highest loading score is shown in Figure 7. The EFA approach for Model 2 identified three latent variables of interest, which included body size (ML1 as BS; Figure 5), ovary size (ML4 as OS; Figure 5), and yield grade (ML1 as YG; Figure 7). These three latent variables were used in subsequent steps for Model 2. Splitting the datasets resulted in four variables that were not associated with any of the latent variables when the EFA network was refined through CFA. Those variables included DENS and IMF, similar to Model 1, as well as uterine horn diameter (UHD) and ribeye area (REA). Both UHD and REA were part of the BC latent variable of Model 1, indicating that splitting the dataset reduced the ability to show their relationship to the YG latent variable in this model.

### 3.3 Confirmatory factor analysis (CFA)

Table 1 demonstrates the two identified latent variables in Model 1 after refining the network provided by EFA by removing cross-loadings and explaining the effect of 14 phenotypic variables (t = 14) with an extent of *R* ^2^ ranging from 0.488 to 0.830 for BS and 0.036 to 0.343 for BC. Similarly, Table 2 demonstrates that the three latent variables identified for Model 2 explained the effect for 12 observed phenotypic parameters (variables, t = 12) after refining the EFA network. The extent of *R* ^2^ ranged from 0.122 to 0.981 for OS, 0.385 to 0.860 for BS, and 0.197 to 0.998 for YG UBT developed in Model 2 of this study. The potential scale reduction factor (PSRF) value of approximately 1 for all model variables proves convergence was met. The extent of *R* ^2^ indicates how strongly each parameter has contributed to the developed UBT. The *R* ^2^ statistics of models also aligned with our factor loading estimates further validating the latent variable structure for underlying biological phenotypes.

**TABLE 1:** Factor loading values for developed underlying biological traits of Model 1 through confirmatory factor analysis and validated by their coefficient of determination (*R* ^2^).

Latent traits	Phenotypic parameters	Loadings	PSD^a^	*R* ^2^
Body Size	Body length	0.699	0.041	0.488
	Body weight	0.901	0.020	0.812
	Flank girth	0.828	0.029	0.686
	Heart girth	0.799	0.032	0.639
	Hip height	0.704	0.044	0.495
	Hip width	0.727	0.039	0.529
	Mid girth	0.911	0.018	0.830
Body Composition	Antral follicle count	0.524	0.087	0.275
	Left ovary diameter	0.561	0.083	0.314
	Ribeye area	0.560	0.083	0.313
	Right ovary diameter	0.513	0.088	0.263
	Rump fat	0.189	0.102	0.036
	Uterine horn diameter	0.586	0.081	0.343
	Yield grade	0.212	0.102	0.045

^a^ PSD, represents the posterior standard deviations.

**TABLE 2:** Factor loading values for developed underlying biological traits of Model 2 through confirmatory factor analysis and validated by their coefficient of determination (*R* ^2^).

Latent traits	Phenotypic paramters	Loadings	PSD^a^	*R* ^2^
Ovary Size	Antral follicle count	0.990	0.016	0.981
	Left Ovary Diameter	0.803	0.051	0.125
	Right Ovary Diameter	0.879	0.052	0.122
Body Size	Body Length	0.720	0.030	0.519
	Body Weight	0.803	0.023	0.644
	Heart Girth	0.879	0.016	0.772
	Hip Height	0.621	0.038	0.385
	Hip Width	0.744	0.028	0.553
	Mid Girth	0.928	0.012	0.860
Yield Grade	Rib Fat	0.999	0.001	0.998
	Rump Fat	0.444	0.064	0.197
	Yield grade	0.999	0.001	0.998

^a^ PSD, represents the posterior standard deviations.

### 3.4 Bayesian network learning analysis

**FIGURE 8:**
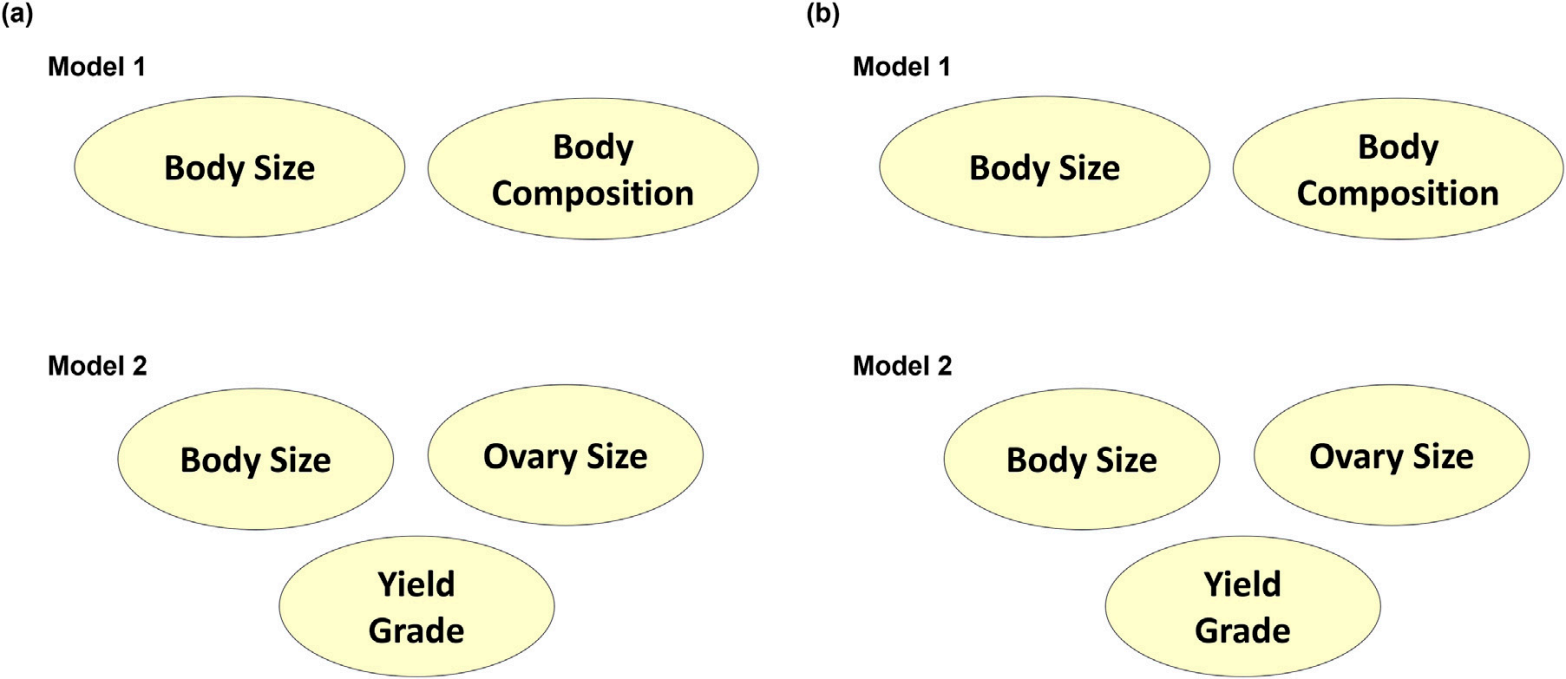
Bayesian networks learned from **(A)** Tabu search algorithm and **(B)** Max-Min Hill-Climbing algorithm to explain interrelationships among composite phenotypes from Models 1 and 2.

**FIGURE 9:**
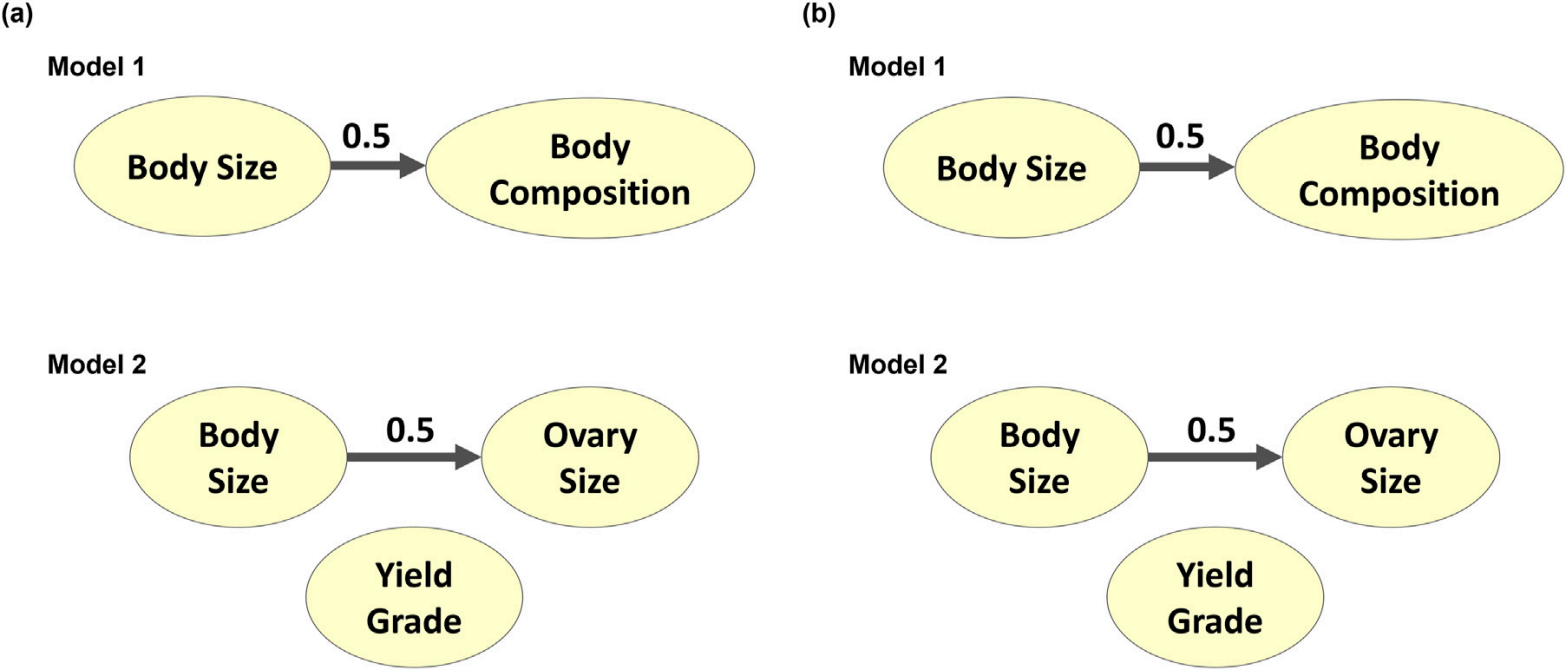
Genotypically adjusted Bayesian networks learned from **(A)** Tabu search algorithm and **(B)** Max-Min Hill-Climbing algorithm to explain corrected interrelationships among composite phenotypes from Models 1 and 2.

The latent variables produced from CFA in both models were then processed through Bayesian network learning algorithms to identify latent variable interrelationships per model. The two algorithms used, Tabu and Max-Min Hill Climbing, showed similar results (Figure 8). The BS and BC traits from Model 1 did not contribute to each other. Similarly, BS from Model 2 did not contribute to OS. Since carcass traits were modeled separately of size and reproductive attributes and only yielded one latent variable, there were no Bayesian networks established for the carcass latent variable identified as YG in Model 2. When the BNL structure was run using the gEBV, BS contributed to BC with a directional signal of 0.5 (minimum threshold) in Model 1, and BS contributed to OS with a directional signal of 0.5 (minimum threshold) for Model 2 (Figure 9).

## 4 Discussion

Two approaches (Model 1 and Model 2) in this study identified 2 and 3 latent variables, respectively, summarizing BS, BC, OS and YG as attributes of growth and size in admixed beef heifers. Research and advances in technology have led to a lot of potential characteristics that producers can capture in animals (Koltes et al., 2019). In this study, body size, body composition, and ovary size latent variables relate to one overarching phenotype–size. Given these size attributes and *R* ^2^ from CFA, producers can capture most of the size variation by taking body weight and girth measures into account. Girth measures have not been a trait collected by producers, but body weight measures have been encouraged in cattle for many years (Cundiff et al., 2018). Among body size-related traits, body density stood alone from latent traits in both models, suggesting that it should be modeled through univariate genome-wide analysis considering its significance as an alternative to body condition score (Li et al., 2022). For reproductive efficiency, AFC is found as a measure of dependency related to size, specifically considering ovary characteristics from Model 2 as also implied earlier (Cushman et al., 2009). Considering the consumer aspects and market demand for the beef production system, beef is the end product and its post-pandemic demands are changing as well (Cowley, 2021). Producers can rely on the overall yield grade as an important contributing parameter to carcass characteristics amidst the production pressures to meet market needs. On the other hand, intramuscular fat (IMF) stood alone irrespective of the model approach, indicating this trait should be another parameter to consider along with yield grade for carcass attributes. Although there are traits producers could or have feasibly captured on their animals, this study does show that additional phenotypes influence these latent variables and have the potential to impact genome-wide analysis outcomes. Therefore, exact phenotypes to recommend to producers for phenotypic capture must come after genome-wide analysis has been conducted, which will be reported in the next paper.

Advancement in handling high-throughput phenotypic data in beef production requires the adaptation of methodologies like factor analysis to handle messy and highly correlated datasets (Koltes et al., 2019; Yu et al., 2020a). The interplay of factor analysis and BNL in this study demonstrated the structure of UBT and how these traits needed to be structured in genome-wide analysis, including which original variables should be focused on univariately without UBT. The BNL structure based on phenotypic data alone was not as accurate due to sample size and lack of genomic relationship information. When using gEBV, the BNL structure changed for both Model 1 and 2, providing enough information to identify the two latent variables’ relationship for an enhanced multi-trait genome-wide approach even with a smaller sample size. Therefore, it is better to confirm the BNL structure after including genotypic data before conducting association analyses (Töpner et al., 2017).

Data pre-filtering for factor analysis sample adequacy is also very important in this approach. Priority in this study was placed on measures captured directly on the animal over calculated measures to ensure feasibility for producer implementation. Outcomes of this study could have been different if calculated measures were used instead. Even so, practicality of phenotypic generation should always drive research in production agriculture, which is why this study did not prioritize calculated measures over direct animal measures. Furthermore, we applied a high correlation threshold (*r* ^2^ ≥ 0.85) as part of the filtering process to cope with confounding traits while still allowing traits the producer could capture to be included. This changed which variables were present between Model 1 and Model 2, although many were the same (t = 15). Furthermore, Model 1 approach was completely data-driven with all possible variables present through the filtering process, whereas Model 2 was semi-subjective given the separation of carcass characteristics from other attributes to account for the sample size difference. Through this, it became evident that a possible relationship was lost in Model 2 compared to Model 1. Therefore, careful planning and pre-processing of data in future applications of factor analysis research should be present.

## Data Availability

The datasets presented in this article are not readily available because of on-going research efforts. Requests to access the datasets should be directed to the corresponding author.
