# Supplementary material for: Multi-trait phenotypic modeling through factor analysis and bayesian network learning to develop latent reproductive, body conformational, and carcass-associated traits in admixed beef heifers
Source: Front Genet. 2025 Mar 24;16:1551967. doi: 10.3389/fgene.2025.1551967 (PMC11973389; doi:10.3389/fgene.2025.1551967)
Supplement: Supplementary file 3 [file DataSheet1.PDF]

```
# Load Packages
library(data.table)
library(readxl)
library(pheatmap)
library(dplyr)
library(tidyr)
library(knitr)
library(MTM)
library(psych)
library(blavaan)
library(bnlearn)
library(AGHmatrix)
library(Rgraphviz)
library(lavaan)
library(coda)
library(utils)
library(qqman)
#########################################
# 1. import data from factor analysis directory and read data
setwd("path/to/Factor_Analyses")
data=read_excel("Phenotype_Data.xlsx", col_names = T)
dim(data) #336  49
##################################################
#Model1: With all paramters at once, data-driven approach
##################################################
sub_data=data[-c(2:9)]
str(sub_data)#checking variable type
sub_data=lapply(sub_data[-1], as.numeric) #changing everything to numeric except sample ID
sub_data$ID=data$ID; sub_data=as.data.frame(sub_data)
sub_data_filtered=na.omit(sub_data) #removing NAs
dim(sub_data_filtered) #159  41
boxplot(sub_data_filtered[, -41]) #each column seems to contain multiple extreme values so needed to check if there are outliers based on standard criteria of upper and lower boundary

#creating function to remove outliers based on Tukey’s outlier rule (Iglewicz, 2011) explained in manuscript.
remove_outliers <- function(data, columns) {
  # based on standard upper and lower boundary of the box plot,
  # the function filters for data points within the range Q1 - 1.5 * IQR and Q3 + 1.5 * IQR
  filtered_data <- data %>%
    filter(if_any(all_of(columns), ~ . > quantile(., 0.25) - 1.5 * IQR(.) & . < quantile(., 0.75) + 1.5 * IQR(.)))
  return(filtered_data)
}

columns_for_outliers <- colnames(sub_data_filtered)[-41]  # defining parameters for outlier filtering except sample ID
sub_data_filtered <- remove_outliers(sub_data_filtered, columns_for_outliers)  # filtering for outliers based on criteria defined earlier
dim(sub_data_filtered) #159  41 #seems like all values fall under criteria

################################################
# Exploratory Factor Analysis with Model 1
################################################
colnames(sub_data_filtered) <- sub("^i", "", colnames(sub_data_filtered))
COR <- cor(sub_data_filtered[,names(sub_data_filtered)!='ID'], use = "complete.obs") # The warning message "the standard deviation is zero" indicates that there is no variation in at least one of the variables used in the cor() function.
#Lets find which one is that
constant_columns <- sapply(sub_data_filtered, function(col) length(unique(col)) == 1) #seems like 'Rxlrg'
# Combine the column names to exclude into a single vector
cols_to_exclude <- c("ID", names(sub_data_filtered)[constant_columns])

# Calculate correlation, excluding constant columns and the 'ID' column
COR <- cor(sub_data_filtered[, !(names(sub_data_filtered) %in% cols_to_exclude)], use =
```

```
"complete.obs")
heatmap3::heatmap3(COR)
KM <- KMO(COR) #matrix seems not invertible so need to filter variables
# a indicator to the sampling adequacy, over 0.3 is acceptable
View(COR)
#lets check for highly correlated traits
cor_threshold <- 0.85

# Find pairs of highly correlated traits (upper triangular part of the correlation matrix)
highly_correlated_pairs <- which(abs(COR) > cor_threshold & upper.tri(COR), arr.ind = TRUE)
# Create a list to store the names of highly correlated traits to remove
traits_to_remove <- character(0)
# Loop through the pairs and identify the traits to remove
for (i in 1:nrow(highly_correlated_pairs)) {
  row_trait <- rownames(COR)[highly_correlated_pairs[i, 1]]
  col_trait <- colnames(COR)[highly_correlated_pairs[i, 2]]
  traits_to_remove <- union(traits_to_remove, col_trait)
}
traits_to_remove
# Remove highly correlated traits from the data
sub_data_filtered_without_corr <- sub_data_filtered[, !(names(sub_data_filtered) %in% traits_to_remove)]
dim(sub_data_filtered_without_corr) #159  30
names(sub_data_filtered_without_corr)

# Calculate the new correlation matrix after removing highly correlated traits
COR_without_corr <- cor(sub_data_filtered_without_corr[, -30], use = "complete.obs")# The warning message "the standard deviation is zero" indicates that there is no variation in at least one of the variables used in the cor() function.
#Lets find which one is that
constant_columns <- sapply(sub_data_filtered_without_corr, function(col) length(unique(col)) == 1) #seems like 'Rxlrg', again
# Combine the column names to exclude into a single vector, most of the paramters included are having their effect explained based on calculated paramters
cols_to_exclude <- c('ID','med', 'Lmed', 'Llrg', 'Lxlrg', 'Rmed', 'Rlrg', 'lrg', 'LOL', 'LOH', 'ROL', 'ROH', 'AOD',names(sub_data_filtered_without_corr)[constant_columns])
sub_data_filtered_without_corr_corrected=sub_data_filtered_without_corr[, !(names(sub_data_filtered_without_corr) %in% cols_to_exclude)]

# Calculate the new correlation matrix after removing highly correlated and already explained traits
COR <- cor(sub_data_filtered_without_corr_corrected[-17], use = "complete.obs")
KM1=KMO(COR)
KM1 # sample adequacy of 0.63 (>= 0.5) indicates that we are good to go for factor analysis

# Running parallel ananlysis to find the number of latent factors for exploratory factor ananlysis

fa.parallel(COR, n.obs = 159, fa = "fa", n.iter = 1000, error.bars = FALSE, # change the n.obs when dataset changes
            se.bars = FALSE, ylabel = 'Eigen values of factors', fm = 'ml') # parallel analysis

efa.fit <- fa(COR, nfactors = 4, rotate = "varimax", fm = "ml", max.iter = 1000) # change nfactors when dataset changes based on parallel analysis results
load <- efa.fit$loadings

# creating figure 2
figure2=pheatmap(load, display_numbers = TRUE, cluster_cols = FALSE, angle_col = 0,
                 main = '', fontsize_number = 10, fontsize = 10) # draw a heatmap with observed phenotypes
#ggsave(filename="figure2.tiff",plot=figure2, units="px", width = 1500, height = 1000,dpi=300)
col_name2=c("ML1 = Body Size", "ML4 = Body Composition","","BWT = Body Weight", "BL = Body Length", "HH = Hip Height", "HW = Hip Width", "HG = Heart Girth", "MG = Mid Girth", "FG = Flank Girth", "DENS = Body Density", "IMF = Intramuscular Fat", "REA = Rib Eye
```

```
Area", "YG = Yield Grade", "RMP = Rump Fat", "UHD = Uterine Horn Diameter", "AFC = Antral Follicular Count", "LOD = Left Ovarian Diameter", "ROD = Right Ovarian Diameter")

# creating figure 3

figure3=fa.diagram(efa.fit, simple=T, cut=0.3, main = "",e.cex=16) # draw a diagram between the observed phenotypes

legend(x=11,y=14, legend = col_name2)
tiff(filename="figure3.tiff", units="px", width = 2500, height = 2000,res =300)
fa.diagram(efa.fit, simple=T, cut=0.3, main = "",e.cex=10) # draw a diagram between the observed phenotypes
legend(x=11,y=16, legend = col_name2)
dev.off()
#####################################
# Confirmatory Factor Analysis of Model 1
#####################################
CFA.Model1 = '# this is the model 1 of body data
# 7
body =~ iMG + iBWT + iFG + iHG + iHW + iHH + iBL
# 7
ReproCarcass =~ UHD + iREA + LOD + iYG + iRMP + ROD + AFC
'

# run this code and update based on your data, for this study lead the file provided as sample
#CFA.fit <- bcfa(CFA.Model1, data = sub_data_filtered_without_corr_corrected[,names(sub_data_filtered_without_corr_corrected) !='ID'],
#                burnin=5000, sample=10000, target="stan",
#                save.lvs=T, n.chains=2, std.lv=TRUE)
#summary(CFA.fit, standardized=TRUE,rsquare=TRUE) # check R_hat
#plot(CFA.fit, plot.type = "trace") # check trace plots
#saveRDS(CFA.fit, file = 'Complete_CFAfit1_burnin_5000_sample_10000.rds')
CFA.fit <- readRDS('Complete_CFAfit1_burnin_5000_sample_10000.rds')

#Table 1 statistics of manuscript

standardizedSolution(CFA.fit, se=T) %>%
  filter(op == "=~") %>%
  select('Latent Factor'=lhs, 'Observed Phenotypes'=rhs,
         'Estimate'=est.std, 'Posterior standard deviation'=se) %>%
  knitr::kable(digits = 3, booktabs=TRUE, format="markdown", caption="Factor Loadings") # a table with some variables
summary(CFA.fit, standardized=TRUE,rsquare=TRUE) # check R_hat
bfs_all <- blavInspect(CFA.fit, 'lvmeans') # get posterior means
colnames(bfs_all) <- c('Body Size', 'Body Composition') # rename columns for body data

#####################################
# Bayesian Network Learning Based on estimated Latent Variables only for model 1
#####################################
# we found relationship in body data, we didn't find relationship in carcass data
check_boot <- function(boot, Strength_Thres, Direction_Thres) {
  boot[(boot$strength >= Strength_Thres) & (boot$direction > Direction_Thres),]
}

# Bredding Value based BLUP phenotypic model
BV=as.data.frame(bfs_all)
colnames(BV) <- colnames(bfs_all)

#tabu-Figure 8
par(mfrow=c(1,2))
tabu_simple <- tabu(BV)
graphviz.plot(tabu_simple, main = "(a)", shape = "ellipse", layout = "dot")

#Max-Min Hill Climbing-Figure 8
mmhc_simple <- mmhc(BV)
graphviz.plot(mmhc_simple, main = "(b)", shape = "ellipse", layout = "dot")
```

```
######################################
# Multi-trait gBLUP and Bayesian Network Learning for model 1
######################################

#to include ID for further work here
cols_to_exclude <- c('med', 'Lmed', 'Llrg', 'Lxlrg', 'Rmed', 'Rlrg', 'lrg', 'LOL', 'LOH', 'ROL', 'ROH', 'AOD',names(sub_data_filtered_without_corr)[constant_columns])
sub_data_filtered_without_corr_corrected=sub_data_filtered_without_corr[, !(names(sub_data_filtered_without_corr) %in% cols_to_exclude)]

phe <- sub_data_filtered_without_corr_corrected #phenotypic data
# After including genotypes

geno <- fread('genotype_filtered2.txt', header = T, check.names=F) # genomic data is not included but will be available on request
dim(geno)
geno <- geno[geno$ID %in% phe$ID, ]
phe <- phe[match(geno$ID, phe$ID), ]
table(geno$ID == phe$ID) # check if they are matching
geno1 <- subset(geno, select = -c(ID))
geno1 <- as.data.frame(lapply(geno1, as.numeric))
geno1 <- as.matrix(geno1)
grm <- Gmatrix(geno1, method = 'VanRaden', maf = 0.05) # Genomic relationship matrix as described in manuscript

## MTM function redefined for gBLUP
MTM_func<- function(X, Y, G, nTr, nIter, burnIn, thin, prefix){
  library(MTM)
  set.seed(2021)
  MTM (
    XF = X,
    Y = Y,
    K = list(
      list(
        K = G,
        COV = list(
          type = 'UN',
          df0 = nTr,
          S0 = diag(nTr)
        )
      )
    ),
    resCov = list(
      type = 'UN',
      S0 = diag(nTr),
      df0 = nTr
    ),
    nIter = nIter,
    burnIn = burnIn,
    thin = thin,
    saveAt = prefix
  )
}


MTM.cfa <- MTM_func(X, Y, G = grm, nTr = ncol(Y),
                    nIter = 90000,burnIn = 10000,
                    thin = 2, 'complete_MTM_pred_geno_noFSG')
list.files(pattern = 'complete_MTM_pred_geno_noFSG')
str(MTM.cfa)
diag(MTM.cfa$K[[1]]$G) # Posterior mean of the covariance matrix
#saveRDS(MTM.cfa, file = 'complete_MTM_pred_geno_noFSG')
#MTM.cfa <- readRDS('complete_MTM_pred_geno_noFSG') #to load the sample saved file

# Bredding Value based on gBLUP model
BV <- MTM.cfa$K[[1]]$U
BV <- as.data.frame(BV)
```

```r
Linv <- solve(t(chol(grm))) # we do this to eliminate the sample dependencies as described by (Töpner et al., 2017; Momen et al., 2021)
Minv <- kronecker(diag(ncol(BV)), Linv)
BV_adj <- matrix(Minv %*% c(as.matrix(BV)), nrow = 159, ncol = 2) # change nrow and ncol accordingly
BV_adj <- as.data.frame(BV_adj)
colnames(BV_adj) <- colnames(MTM.cfa$YHat)

#tabu-Figure 9
par(mfrow=c(1,2))
tabu_simple <- tabu(BV_adj); tabu_simple #branching factor of 0.5 indicate the directional signal
graphviz.plot(tabu_simple, main = "(a)", shape = "ellipse", layout = "dot")


#Max-Min Hill Climbing-Figure 9
mmhc_simple <- mmhc(BV_adj); mmhc_simple #branching factor of 0.5 indicate the directional signal
graphviz.plot(mmhc_simple, main = "(b)", shape = "ellipse", layout = "dot")

################################################################################################################################################################
#Model2:With Split Data
################################################################################################################################################################
# Body Conformational here
body_data=data[-c(22:26)]
dim(body_data)
names(body_data)
str(body_data)#checking variable type
body_sub_data=lapply(body_data[-(1:9)], as.numeric)
body_sub_data$ID=body_data$ID; body_sub_data=as.data.frame(body_sub_data)
dim(body_sub_data)
body_sub_data_filtered=na.omit(body_sub_data) #removing NAs
dim(body_sub_data_filtered) #298  36
par(mfrow=c(1,1))
boxplot(body_sub_data_filtered[, -36]) #each column seems to contain multiple extreme values so needed to check if there are outliers based on standard criteria of upper and lower boundary

columns_for_outliers <- colnames(body_sub_data_filtered)[-36]  # defining parameters for outlier filtering
body_sub_data_filtered <- remove_outliers(body_sub_data_filtered, columns_for_outliers) # filtering for outliers based on criteria defined earlier
dim(body_sub_data_filtered) #298  36 #seems like all values fall under criteria

###################################################################
#Exploratory Factor Analysis with Body Conformational Parameters
###################################################################
colnames(body_sub_data_filtered)=sub("^i", "", colnames(body_sub_data_filtered))
COR <- cor(body_sub_data_filtered[,names(body_sub_data_filtered)!='ID'], use = "complete.obs") # The warning message "the standard deviation is zero" indicates that there is no variation in at least one of the variables used in the cor() function.
#View(COR)
KM = KMO(COR)  #matrix is not invertible

#lets check for highly correlated traits
cor_threshold <- 0.85

# Find pairs of highly correlated traits (upper triangular part of the correlation matrix)
highly_correlated_pairs <- which(abs(COR) > cor_threshold & upper.tri(COR), arr.ind = TRUE)
# Create a list to store the names of highly correlated traits to remove
traits_to_remove <- character(0)
# Loop through the pairs and identify the traits to remove
for (i in 1:nrow(highly_correlated_pairs)) {
  row_trait <- rownames(COR)[highly_correlated_pairs[i, 1]]
  col_trait <- colnames(COR)[highly_correlated_pairs[i, 2]]
```

```
  traits_to_remove <- union(traits_to_remove, col_trait)
}
traits_to_remove  #highly correlated traits to be removed from body_sub_data_filtered
# Remove highly correlated traits from the data
body_sub_data_filtered_without_corr <- body_sub_data_filtered[, !(names(body_sub_data_filtered) %in% traits_to_remove)]
dim(body_sub_data_filtered_without_corr) #298  23
names(body_sub_data_filtered_without_corr)
# Calculate the new correlation matrix after removing highly correlated traits
COR_without_corr <- cor(body_sub_data_filtered_without_corr[, -29], use = "complete.obs")# The warning message "the standard deviation is zero" indicates that there is no variation in at least one of the variables used in the cor() function.
KM2=KMO(COR_without_corr) #matrix is not invertible
#Lets find if someone is having no or constant standard deviation
constant_columns <- sapply(body_sub_data_filtered_without_corr, function(col) length(unique(col)) == 1)
# Combine the column names to exclude into a single vector, most of the paramters included are having their effect explained based on calculated paramters
cols_to_exclude <- c('Lmed', 'Llrg', 'Lxlrg', 'Rmed', 'Rlrg','xlrg', 'Rxlrg', 'lrg', 'LOL', 'LOH', 'ROL', 'ROH','AOL','AOH', 'AOD', 'Rsm', 'med',names(body_sub_data_filtered_without_corr)[constant_columns])

body_sub_data_filtered_without_corr=body_sub_data_filtered_without_corr[, !(names(body_sub_data_filtered_without_corr) %in% cols_to_exclude)]
COR <- cor(body_sub_data_filtered_without_corr[-12], use = "complete.obs")

dim(body_sub_data_filtered_without_corr)

KM3=KMO(COR);KM3 # sample adequacy of 0.57 (>= 0.5) indicates that we are good to go for factor analysis

# running parallel analysis to find the number of factors as described earlier
fa.parallel(COR, n.obs = 298, fa = "fa", n.iter = 1000, error.bars = FALSE, # change the n.obs when dataset changes
            se.bars = FALSE, ylabel = 'Eigen values of factors', fm = 'ml') # parallel analysis

# running the factor analysis to find the number of underlying traits
efa.fit1 <- fa(COR, nfactors = 5, rotate = "varimax", fm = "ml", max.iter = 1000) # change nfactors when dataset changes
load1 <- efa.fit1$loadings
figure4=pheatmap(load1, display_numbers = TRUE, cluster_cols = FALSE, angle_col = 0,
                 main = '', fontsize_number = 10, fontsize = 10) # draw a heatmap with observed phenotypes

ggsave(filename="figure4.tiff",plot=figure4, units="px", width = 1500, height = 1000,dpi=300)
tiff(filename="figure5.tiff", units="px", width = 2500, height = 2000,res =300)
fa.diagram(efa.fit1, simple=T, cut=0.3, main = "") # draw a diagram between the observed phenotypes
col_name4=c("ML1 = Body Size", "ML4 = Ovary Size", "","BWT = Body Weight", "BL = Body Length", "HH = Hip Height", "HW = Hip Width", "HG = Heart Girth", "MG = Mid Girth", "DENS = Body Density", "UHD = Uterine Horn Diameter", "AFC = Antral Follicular Count", "LOD = Left Ovarian Diameter", "ROD = Right Ovarian Diameter")
legend(x=7,y=10, legend = col_name4)
dev.off()


#####################################
# Confirmatory Factor Analysis for Model 2 body size related data
#####################################
CFA.Model2 = '# this is the model of body data
# 6
body =~ iMG + iBWT + iHG + iHW + iHH + iBL
# 7
ovary =~ AFC + LOD + ROD
'
#Run the model for your data based on factor analysis of your data or load the sample data from this study
```

```
#CFA.fit2 <- bcfa(CFA.Model2, data = body_sub_data_filtered_without_corr[,names(body_sub_data_filtered_without_corr)!='ID'],
#                burnin=5000, sample=10000, target="stan",
#                save.lvs=T, n.chains=2, std.lv=TRUE)
#summary(CFA.fit2, standardized=TRUE,rsquare=TRUE) # check R_hat
#plot(CFA.fit2, plot.type = "trace") # check trace plots
#saveRDS(CFA.fit2, file = 'body_ovary_CFAfit1_burnin_5000_sample_10000.rds')
CFA.fit2 <- readRDS('body_ovary_CFAfit1_burnin_5000_sample_10000.rds') # loading sample data of stduy
#Table 2 statistics
standardizedSolution(CFA.fit2, se=T) %>%
  filter(op == "=~") %>%
  select('Latent Factor'=lhs, 'Observed Phenotypes'=rhs,
         'Estimate'=est.std, 'Posterior standard deviation'=se) %>%
  knitr::kable(digits = 3, booktabs=TRUE, format="markdown", caption="Factor Loadings") # a table with some variables

bfs_all2 <- blavInspect(CFA.fit2, 'lvmeans') # get posterior means
colnames(bfs_all2) <- c('Body Size', 'Ovary Size') # rename columns for body data


#####################################
#Bayesian Network Learning for Model 2 Based on estimated Latent Variables only of body size related trait
#####################################
# we found relationship in body data, we didn't find relationship in carcass data
check_boot <- function(boot, Strength_Thres, Direction_Thres) {
  boot[(boot$strength >= Strength_Thres) & (boot$direction > Direction_Thres),]
}

BV1 <- bfs_all2
BV1 <- as.data.frame(BV1)

colnames(BV1) <- colnames(bfs_all2)

#tabu - Figure 8
tabu_simple1 <- tabu(BV1)
graphviz.plot(tabu_simple1, main = "(a)", shape = "ellipse", layout = "dot")

#Max-Min Hill Climbing - Figure 8
mmhc_simple1 <- mmhc(BV1)
graphviz.plot(mmhc_simple1, main = "(b)", shape = "ellipse", layout = "dot")

#####################################
# Multi-trait gBLUP and Bayesian Network Learning for model 2
#####################################


# After including genotypes
phe1 <- body_sub_data_filtered_without_corr
geno <- fread('genotype_filtered2.txt', header = T, check.names=F) # genomic data is not included but will be available on request
geno[1:10,1:10]
colnames(geno)[1] <- "ID"
geno2 <- geno[geno$ID %in% body_sub_data_filtered_without_corr$ID, ]
phe1 <- body_sub_data_filtered_without_corr[match(geno2$ID, body_sub_data_filtered_without_corr$ID), ]
table(geno2$ID == phe1$ID) # check if they are matching
geno3 <- subset(geno2, select = -c(ID))
geno3 <- as.data.frame(lapply(geno3, as.numeric))
geno3 <- as.matrix(geno3)
grm1 <- Gmatrix(geno3, method = 'VanRaden', maf = 0.05) # Genomic relationship matrix as described earlier

## MTM function redefined for gBLUP
MTM_func<- function(X, Y, G, nTr, nIter, burnIn, thin, prefix){
  library(MTM)
  set.seed(2021)
  MTM (
```

```
    XF = X,
    Y = Y,
    K = list(
      list(
        K = G,
        COV = list(
          type = 'UN',
          df0 = nTr,
          S0 = diag(nTr)
        )
      )
    ),
    resCov = list(
      type = 'UN',
      S0 = diag(nTr),
      df0 = nTr
    ),
    nIter = nIter,
    burnIn = burnIn,
    thin = thin,
    saveAt = prefix
  )
}

Y1 <- data.frame(scale(bfs_all2))

fphe <- data[c(1:9)] # import fixed effects
fphe1 <- fphe[fphe$ID %in% phe1$ID, ]
fphe1[which(fphe1$Year =="2014"),"Yr"] = 1 # change it to birth year when using carcass data
fphe1[which(fphe1$Year =="2015"),"Yr"] = 2
fphe1[which(fphe1$Year =="2016"),"Yr"] = 3
fphe1[which(fphe1$Year =="2017"),"Yr"] = 4

X1 <- model.matrix(~ -1 + as.factor(fphe1$Yr)+ as.factor(fphe1$Gen) + as.factor(fphe1$DA)
                   + as.factor(fphe1$PBG)) # making as fixed effects #  removed as their is only one generation in this dataset
MTM.cfa1 <- MTM_func(X1, Y1, G = grm1, nTr = ncol(Y),
                    nIter = 90000,burnIn = 10000,
                    thin = 2, 'body_ovary_MTM_pred_geno_test_noFSG.rds')
list.files(pattern = 'body_ovary_MTM_pred_geno_test_noFSG.rds')
str(MTM.cfa1)
diag(MTM.cfa1$K[[1]]$G) # Posterior mean of the covariance matrix
#saveRDS(MTM.cfa, file = 'body_ovary_MTM_pred_geno_test_noFSG.rds')
#MTM.cfa1 <- readRDS('body_ovary_MTM_pred_geno_test_noFSG.rds') #to load the sample saved file

# Bredding Value based on gBLUP model
BV1 <- MTM.cfa1$K[[1]]$U
BV1 <- as.data.frame(BV1)
Linv1 <- solve(t(chol(grm1))) # we do this to eliminate the sample dependencies as described earlier
Minv1 <- kronecker(diag(ncol(BV1)), Linv1)
BV_adj1 <- matrix(Minv1 %*% c(as.matrix(BV1)), nrow = 298, ncol = 2) # change nrow and ncol accordingly
BV_adj1 <- as.data.frame(BV_adj1)
colnames(BV_adj1) <- colnames(bfs_all2)
par(mfrow=c(1,2))


#tabu-Figure 9
tabu_simple1 <- tabu(BV_adj1);tabu_simple1  #branching factor of 0.5 indicate the directional signal
graphviz.plot(tabu_simple1, main = "(a)", shape = "ellipse", layout = "dot")

#Max-Min Hill Climbing-Figure 9
mmhc_simple1 <- mmhc(BV_adj1); mmhc_simple1  #branching factor of 0.5 indicate the directional signal
graphviz.plot(mmhc_simple1, main = "(b)", shape = "ellipse", layout = "dot")
```

```
#####################################################################
#Model 2 Carcass Parameters
#####################################################################
carcass_data=data[c(1:9,22:26)]
names(carcass_data)
str(carcass_data)#checking variable type
carcass_sub_data=lapply(carcass_data[-(1:9)], as.numeric)
carcass_sub_data$ID=carcass_data$ID; carcass_sub_data=as.data.frame(carcass_sub_data)
dim(carcass_sub_data) #336   6
carcass_sub_data_filtered=na.omit(carcass_sub_data) #removing NAs
dim(carcass_sub_data_filtered) #161   6
colnames(carcass_sub_data_filtered) <- sub("^i", "", colnames(carcass_sub_data_filtered))

boxplot(carcass_sub_data_filtered[, -6]) #each column seems to contain multiple extreme values so needed to check if there are outliers based on standard criteria of upper and lower boundary

columns_for_outliers <- colnames(carcass_sub_data_filtered)[-6]  # defining parameters for outlier filtering
carcass_sub_data_filtered <- remove_outliers(carcass_sub_data_filtered, columns_for_outliers)  # filtering for outliers based on criteria defined earlier
dim(carcass_sub_data_filtered) #161  6 #seems like all values fall under criteria

##################################################################
#Exploratory Factor Analysis for Model 2 with Carcass Data
##################################################################
COR <- cor(carcass_sub_data_filtered[,names(carcass_sub_data_filtered)!='ID'], use = "complete.obs") # The warning message "the standard deviation is zero" indicates that there is no variation in at least one of the variables used in the cor() function.
KM4 = KMO(COR)  # sample adequacy of 0.59 (>= 0.5) indicates that we are good to go for factor analysis
#Running parallel analsysis to find out the number of factors required for factor analysis
fa.parallel(COR, n.obs = 161, fa = "fa", n.iter = 1000, error.bars = FALSE, # change the n.obs when dataset changes
            se.bars = FALSE, ylabel = 'Eigen values of factors', fm = 'ml') # parallel analysis
#running factor analysis based on parallel analysis results
efa.fit2 <- fa(COR, nfactors = 2, rotate = "varimax", fm = "ml", max.iter = 1000) # change nfactors when dataset changes
load2 <- efa.fit2$loadings
figure6=pheatmap(load2, display_numbers = TRUE, cluster_cols = FALSE, angle_col = 0,
                 main = '', fontsize_number = 10, fontsize = 10) # draw a heatmap with observed phenotypes
ggsave(filename="figure6.tiff",plot=figure6, units="px", width = 1500, height = 1000,dpi=300)
tiff(filename="figure7.tiff", units="px", width = 2500, height = 2000,res =300)
fa.diagram(efa.fit2, simple=T, cut=0.3, main = "") # draw a diagram between the observed phenotypes
col_name6=c("ML1 = Yield Grade","","IMF = Intramuscular Fat", "REA = Rib Eye Area", "YG = Yield Grade", "RIB = Rib Fat", "RMP = Rump Fat")
legend(x=4,y=4, legend = col_name6)
dev.off()
#####################################
#Confirmatory Factor Analysis for Model 2 with Carcass Data
#####################################
CFA.Model3 = '# this is the model of carcass data
# 3
carcass =~ iYG + iRIB + iRMP
'
#Modify and run according to your model or lead the sample data from this study
#CFA.fit3 <- bcfa(CFA.Model3, data = carcass_sub_data_filtered[,names(carcass_sub_data_filtered)!='ID'],
#                 burnin=5000, sample=10000, target="stan",
#                 save.lvs=T, n.chains=2, std.lv=TRUE)
#summary(CFA.fit3, standardized=TRUE,rsquare=TRUE) # check R_hat
#plot(CFA.fit3, plot.type = "trace") # check trace plots
```

```
#saveRDS(CFA.fit3, file = 'carcass_CFAfit3_burnin_5000_sample_10000.rds')
CFA.fit3 <- readRDS('carcass_CFAfit3_burnin_5000_sample_10000.rds')
summary(CFA.fit3, standardized=TRUE,rsquare=TRUE) # check R_hat
# Table 2 statistics
standardizedSolution(CFA.fit3, se=T) %>%
  filter(op == "=~") %>%
  select('Latent Factor'=lhs, 'Observed Phenotypes'=rhs,
         'Estimate'=est.std, 'Posterior standard deviation'=se) %>%
  knitr::kable(digits = 3, booktabs=TRUE, format="markdown", caption="Factor Loadings")
  # a table with some variables

bfs_all3 <- blavInspect(CFA.fit3, 'lvmeans') # get posterior means

# We do not need to run Bayesian network learning for this model as we only have one
underlying biological trait to move forward with.
```
